# Left and right ventricular longitudinal strains are associated with poor outcome in COVID-19: a systematic review and meta-analysis

**DOI:** 10.1186/s40560-020-00519-3

**Published:** 2021-01-12

**Authors:** Arief Wibowo, Raymond Pranata, Astri Astuti, Badai Bhatara Tiksnadi, Erwan Martanto, Januar Wibawa Martha, Augustine Purnomowati, Mohammad Rizki Akbar

**Affiliations:** 1 Department of Cardiology and Vascular Medicine, Faculty of Medicine Universitas Padjadjaran, Rumah Sakit Umum Pusat Hasan Sadikin, Jalan Professor Eyckman No.38, Pasteur, Bandung, Jawa Barat 40161 Indonesia; 2 Faculty of Medicine, Universitas Pelita Harapan, Tangerang, Indonesia

**Keywords:** COVID-19, Echocardiography, SARS-CoV-2, Longitudinal strain, Ventricle

## Abstract

**Background:**

This systematic review and meta-analysis aimed to assess whether ventricular longitudinal strain can be used as a prognostication tool in patients with coronavirus disease 2019 (COVID-19).

**Methods:**

Systematic literature searches of PubMed, Embase, and EuropePMC databases were performed on 16 November 2020. Left ventricular global longitudinal strain (LV-GLS) refers to LV contraction measurement using the speckle tracking-based method refers to the mean of strain values of the RV free wall (three segments) measured using echocardiography. The main outcome was poor outcome, defined as a composite of mortality and severe COVID-19.

**Results:**

Seven studies comprising of 612 patients were included in meta-analysis. Six studies have mortality as their outcome, and 1 study has severity as their outcome. Patients with poor outcome have lower LV-GLS (SMD 1.15 (0.57, 1.72), *p* < 0.001; *I*^2^ 70.4%). Each 1% decrease in LV-GLS was associated with 1.4x increased risk of poor outcome (OR 1.37 (1.12, 1.67), *p* = 0.002; *I*^2^ 48.8%). Patients with poor outcome have lower RV-LS (SMD 1.18 (0.91, 1.45), *p* < 0.001; *I*^2^ 0%). Each 1% decrease in RV-LS was associated with 1.3x increased risk of poor outcome (OR 1.25 (1.15, 1.35), *p* < 0.001; *I*^2^ 11.8%). Subgroup analysis showed that for every 1% decrease in LV-GLS and RV-LS is increased mortality with OR of 1.30 (1.12, 1.50) and OR of 1.24 (1.14, 1.35), respectively.

**Conclusion:**

This study shows that lower LV-GLS and RV-LS measurements were associated with poor outcome in patients with COVID-19.

**Trial registration:**

PROSPERO CRD42020221144

## Introduction

Coronavirus disease 2019 (COVID-19) is spreading rapidly and is one of the most common disease today [1]. Although most of COVID-19 infections are asymptomatic or mildly symptomatic, a proportion of patients, especially those with comorbidities 10.1016/j.archger.2020.104324, are at higher risk for severe illness. Severe illness may lead to cardiac injury, multiple organ failure, and death [2, 3].

Since cardiac injury is frequently encountered in patients with COVID-19, routine echocardiography in selected patients is reasonable. Bedside echocardiography is time and cost-efficient. Routine echocardiography is also useful in critical care settings. Global longitudinal strain (GLS) measurement provides direct measurement of myocardial deformation; thus, it is less affected by different loading conditions compared to ejection fraction [4]. Ventricular strain has been shown to be a useful tool for prognostication in patients with acute respiratory distress syndrome (ARDS) [5]. Studies indicate the potential use of ventricular strain assessment for prognostication in patients with COVID-19 [6–8].

This systematic review and meta-analysis aimed to assess whether ventricular longitudinal strain can be used as a prognostication tool in patients with COVID-19. Ventricular longitudinal strain comprises of left ventricular GLS (LV-GLS) and right ventricular free wall longitudinal strain (RV-LS), measured using speckle tracking echocardiography.

## Methods

**Fig. 1:**
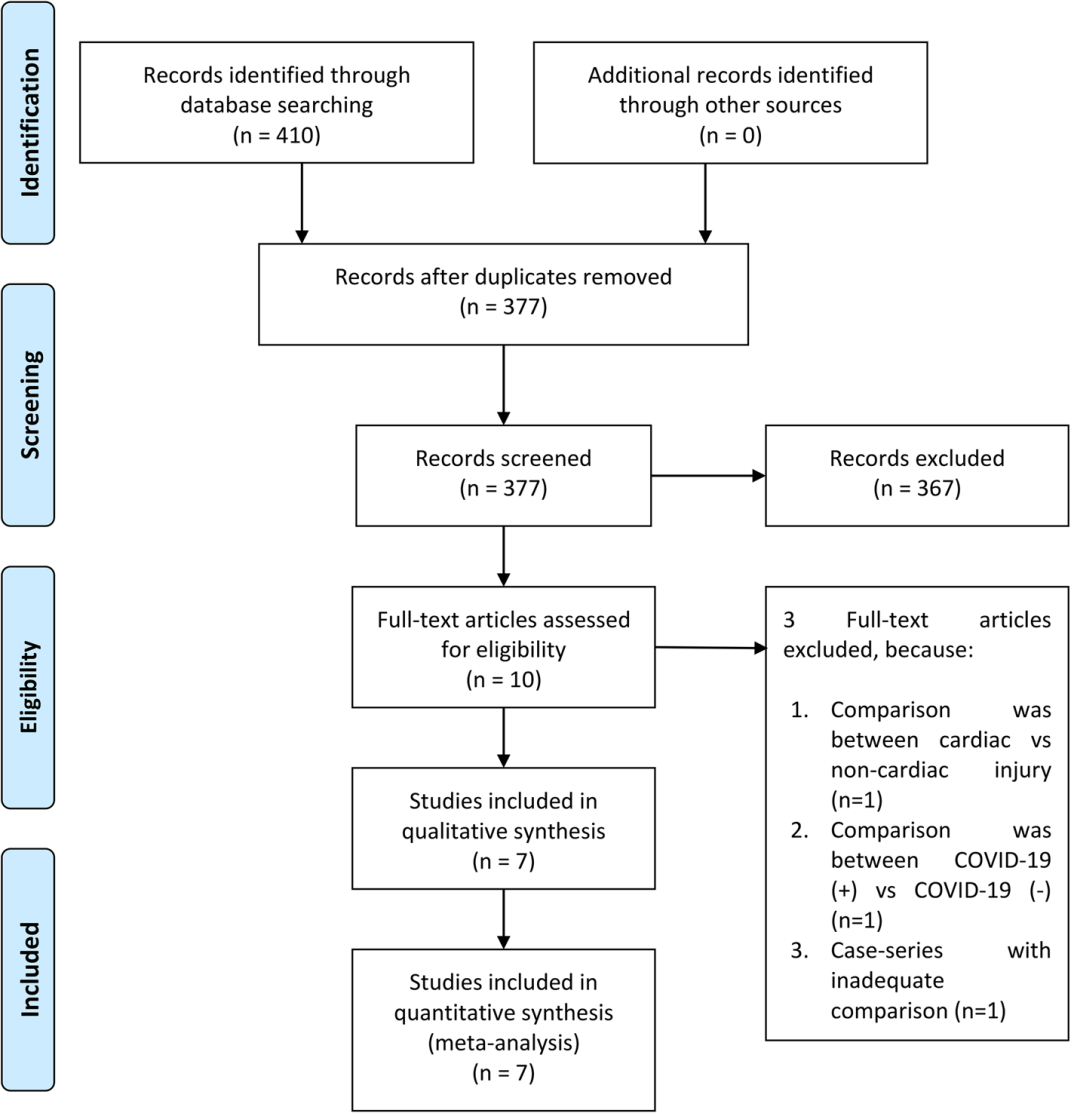
PRISMA flowchart

This study is in accordance with the Preferred Reporting Items for Systematic Reviews and Meta-Analyses (PRISMA) reporting guidelines (Fig. 1). The protocol for this study is registered in the PROSPERO [9] (CRD42020221144).

### Eligibility criteria

The inclusion criteria were all studies with primary data reporting patients with COVID-19 along with the data on the LV-GLS or RV-LS for prognostic purposes. The main outcome was poor outcome, defined as a composite of mortality and severe COVID-19. Mortality was defined as clinically validated death/mortality/non-survivor. Severe COVID-19 was defined as COVID-19 that fulfills the criteria for severe CAP, including the need for mechanical ventilation [10].

The exclusion criteria were preprints, review articles, non-research letters, commentaries, case reports, and articles in non-English Language. Preprints were excluded due to inconsistent credibility [11].

### Search strategy and study selection

Systematic literature searches of PubMed, Embase, and EuropePMC databases were performed with keywords “SARS-CoV-2” OR “COVID-19” OR “2019-nCoV” AND “longitudinal strain” on 16 November 2020. The PubMed (MEDLINE) search strategy was ((SARS-CoV-2) OR (COVID-19) OR (2019-nCoV)) AND (longitudinal strain)). Duplicates were removed, and two authors independently screened the articles’ title/abstract. Potentially relevant articles were then assessed for eligibility based on the inclusion and exclusion criteria.

### Data extraction

Two independent authors extracted the data from eligible studies with the help of standardized extraction form that comprises of first author, year of publication, study design, age, male (gender), hypertension, heart failure, coronary artery disease, chronic lung disease (chronic obstructive pulmonary disease and asthma), the outcome of interest, and its effect estimates.

LV-GLS refers to the measurement of LV contraction using the speckle tracking-based method [12, 13]. RV-LS refers to the mean of strain values of the RV free wall (three segments) measured using echocardiography [14].

The main outcome was poor outcome; the effect estimate was reported in odds ratio (OR) which is pooled from the adjusted effect estimate extracted from the corresponding studies. Standardized mean difference (SMD) of the LV-GLS and RV-LS between patients with poor outcome and favorable outcome was obtained.

The Newcastle-Ottawa Scale (NOS) was used to facilitate quality assessment of the included studies which was performed by two independent authors. Discrepancies that arise at the end of assessment were resolved by discussion.

### Statistical analysis

To perform meta-analysis of the extracted data, we use STATA 16 (StataCorp LLC, Texas, USA). Effect estimate comprising of continuous variables were reported as SMD along with its 95% confidence interval (95% CI). The adjusted ORs were pooled using the DerSimonian & Laird random-effects model, irrespective of heterogeneity, and reported as ORs along with its 95% CI. The *p* values of the effect estimates were two-tailed, and statistical significance was set at ≤ 0.05. To assess inter-study heterogeneity, we performed *I* squared (*I*^2^) and Cochrane Q test; a value of > 50% or *p* value < 0.10 indicates the presence of heterogeneity. Inverted funnel plot analysis was generated to help evaluate the presence of publication bias qualitatively. Regression-based Egger’s test was conducted to assess the presence of small-study effects. We performed a subgroup analysis to assess the association between LV-GLS, RV-GLS, and mortality.

## Results

### Baseline characteristics

Initial search yields 410 records, and 377 remained after removal of duplicates. The articles were then screened for title/abstract, and 367 records were excluded. Ten potentially eligible articles were evaluated based on the inclusion and exclusion criteria. Three full-text articles were excluded because (1) comparison was between cardiac vs non-cardiac injury (*n* = 1); (2) comparison was between COVID-19 (+) vs COVID-19 (-) (*n* = 1); and (3) case series with inadequate comparison (*n* = 1). There were seven studies comprising of 612 patients in the qualitative and quantitative synthesis [4, 6–8, 15–17]. The baseline characteristics of the included studies are displayed in Table 1. Six studies have mortality as their outcome, and 1 study has severity as their outcome. The result of quality assessment using NOS can be seen in Table 1.

**Table 1:** Baseline characteristics of the included studies

Author	Design	Sample	Age (mean/median)	Male (%)	Hypertension (%)	HF (%)	CAD (%)	CLD (%)	Outcome	NOS
Baycan et al. [16]	Retrospective cohort	100	55.6	50	29	-	-	-	Mortality	8
Bursi et al. [17]	Retrospective cohort	49	65.7	63.3	49	6.1	22.4	12.2	Mortality	8
Croft et al. [6]	Retrospective cohort	58	54.1	58.6	68.9	17.2	22.4	13.8	Mortality	6
Janus et al. [7] (letter)	Retrospective cohort	31	64	-	-	-	-	-	Mortality	-
Kim et al. [15]	Prospective–retrospective cohort	40	60	50	37.5	0	0	0.03	Severity	8
Lassen et al. [4]	Prospective cohort	214	68.9	54.7	57	10.3	15.9	15	Mortality	8
Li et al. [8]	Cohort	120	61	48	40	-	9.2	5	Mortality	9

*CAD* coronary artery disease, *CLD* chronic lung disease, *HF* heart failure, *NOS* Newcastle Ottawa Scale

### LV-GLS and poor outcome

**Fig. 2:**
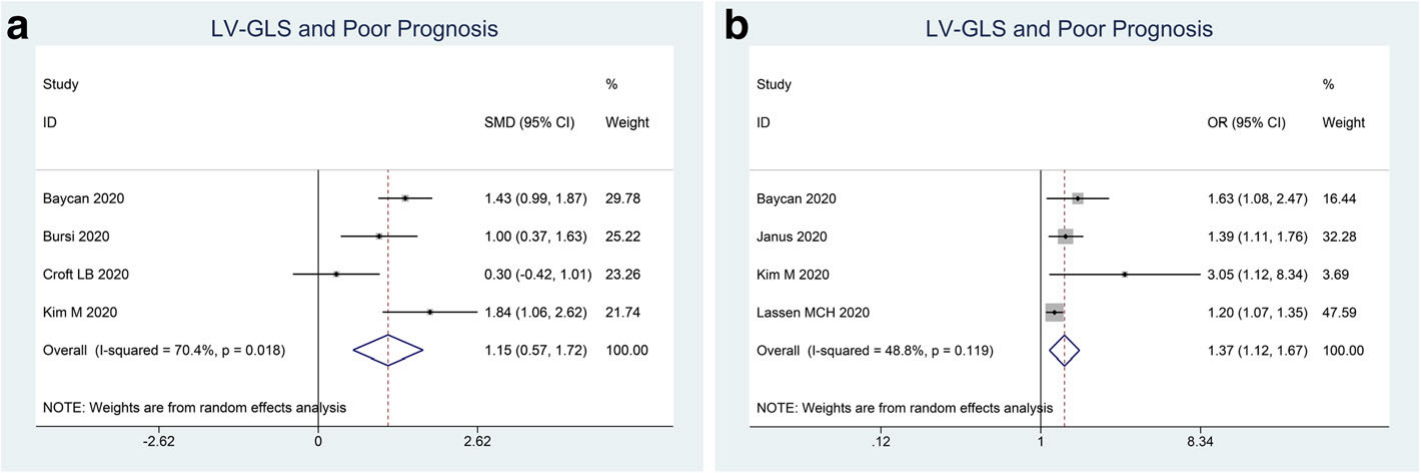
LV-GLS and poor outcome. Mean difference between patients with poor outcome and those without (**a**) and adjusted OR for each 1% decrease in LV-GLS. LV-GLS, left ventricular global longitudinal strain; OR, odds ratio

Patients with poor outcome have lower LV-GLS (SMD 1.15 (0.57, 1.72), *p* < 0.001; *I*^2^ 70.4%, *p* = 0.018) (Fig. 2a). Each 1% decrease in LV-GLS was associated with 1.4x increased risk of poor outcome (OR 1.37 (1.12, 1.67), *p* = 0.002; *I*^2^ 48.8%, *p* = 0.119) (Fig. 2b).

Subgroup analysis for mortality indicated that LV-GLS (SMD 0.96 (0.32, 1.60), *p* < 0.001; *I*^2^ 71.7%, *p* = 0.029) was lower in non-survivors. Each 1% decrease in LV-GLS was associated with 1.3x increased mortality (OR 1.30 (1.12, 1.50), *p* = 0.001; *I*^2^ 30.8%, *p* = 0.236).

### RV-LS and poor outcome

**Fig. 3:**
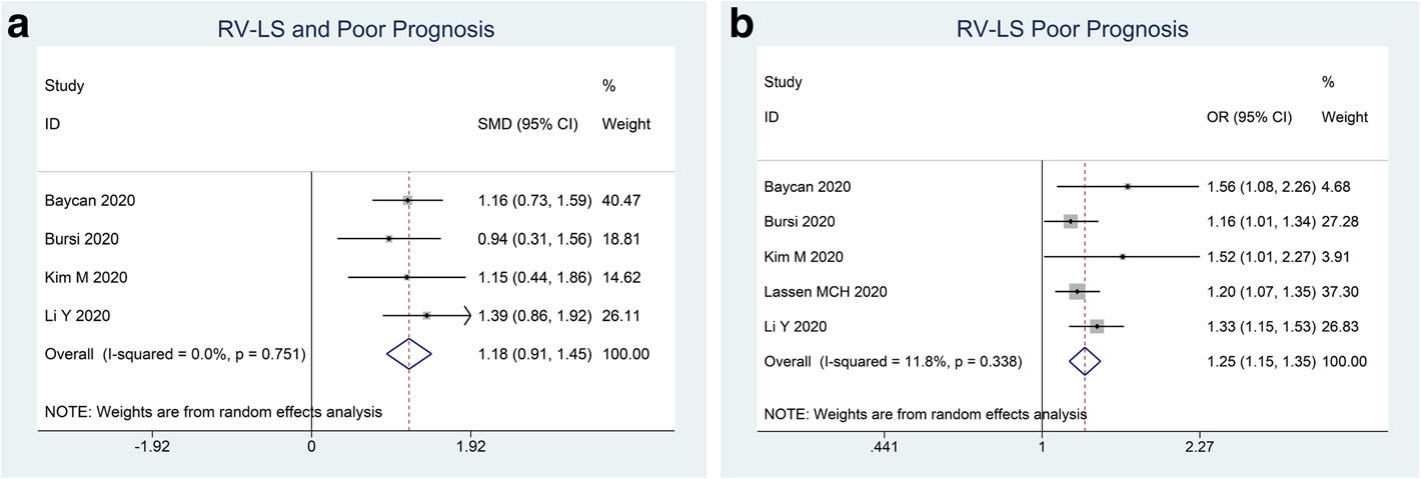
RV-LS and poor outcome. Mean difference between patients with poor outcome and those without (**a**) and adjusted OR for each 1% decrease in RV-LS. RV-LS, right ventricular longitudinal strain, OR, odds ratio

Patients with poor outcome have lower RV-LS (SMD 1.18 (0.91, 1.45), *p* < 0.001; *I*^2^ 0%, *p* = 0.751) (Fig. 3a). Each 1% decrease in RV-LS was associated with 1.3x increased risk of poor outcome (OR 1.25 (1.15, 1.35), *p* < 0.001; *I*^2^ 11.8%, *p* = 0.338) (Fig. 3b).

Subgroup analysis for mortality indicated that RV-LS (SMD 1.18 (0.89, 1.48), *p* < 0.001; *I*^2^ 0%, *p* = 0.549) was lower in non-survivors. Each 1% decrease in RV-LS was associated with 1.2x increased mortality (OR 1.24 (1.14, 1.35), *p* < 0.001; *I*^2^ 15.8%, *p* = 0.313).

### Risk of publication bias

**Fig. 4:**
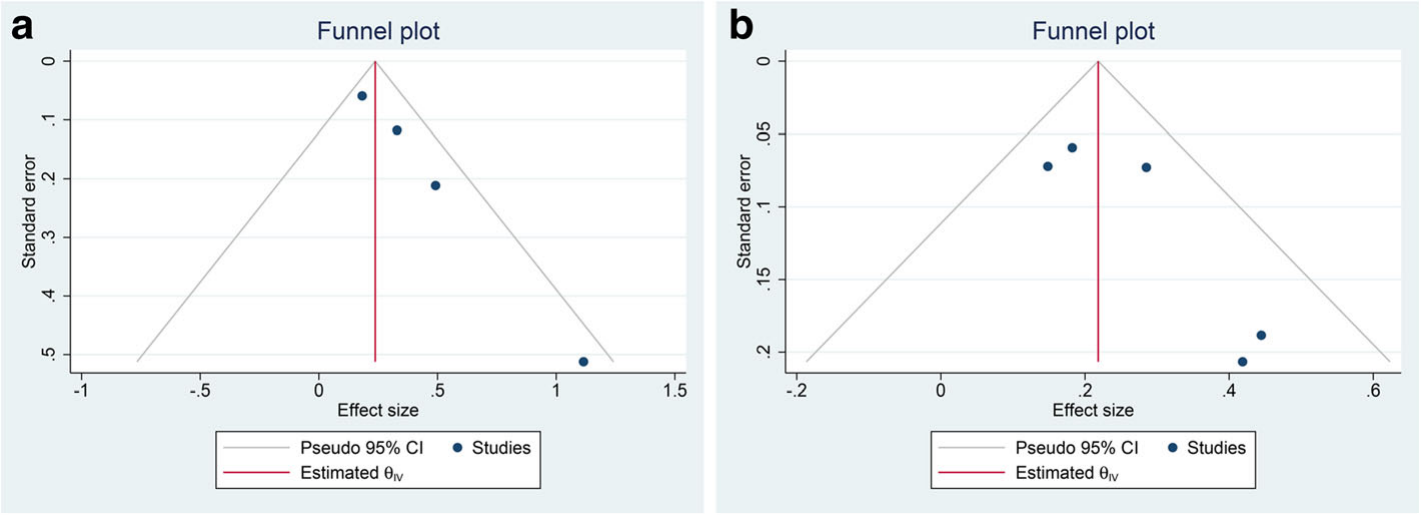
Funnel-plot analysis. LV-GLS (**a**) and RV-LS (**b**). LV-GLS, left ventricular global

The mean difference in terms of LV-GLS in patients with poor outcome and those without having a symmetrical funnel plot and was non-significant for small-study effects (*p* = 0.888). However, pooled OR for LV-GLS has an asymmetrical funnel plot and was significant for small-study effects (*p* = 0.016) (Fig. 4a).

The funnel plot was asymmetrical for the mean difference in terms of RV-LS; however, it was not statistically significant for small-study effects (*p* = 0.062). The pooled OR for RV-LS also has an asymmetrical funnel plot (Fig. 4b) but a not statistically significant for small-study effects (*p* = 0.099).

## Discussion

This meta-analysis indicates that lower LV-GLS and RV-LS were independently associated with poor outcome in COVID-19. Subgroup analysis indicate that for every 1% decrease in LV-GLS and RV-LS, the mortality increased for 1.3x and 1.24x, respectively.

Systemic inflammation induced by COVID-19 may culminate in LV and RV failure [2, 18–21]. Patients with COVID-19 often have reduced basal LV strain, especially in those with obesity, diabetes, and hypertension [22]. The latter comorbidities affect the subendocardial fibers, inducing LV fibrotic process [23, 24]. Lassen et al. also reported lower LV-GLS and RV-LS in patients with COVID-19 compared to those without [4]. Cardiac injury is frequently observed in patients with COVID-19 and is associated with increased mortality [3]. Myocardial injury is expected to affect GLS and GLS has been shown to be a predictor of heart failure in patients with myocardial infarction [25]. However, a study showed that elevated troponin was not associated with LV-GLS in patients with COVID-19, despite an increase in adverse events [26]. A smaller study showed that there was no significant difference in terms of LV-GLS between those without COVID-19, with COVID-19, and COVID-19 + increased cardiac troponin [27]. Thus, reduced GLS might also be affected by acute conditions such as myopericardial damage and acute respiratory distress syndrome to other chronic causes (comorbidities) such as cardiovascular diseases, hypertension, and diabetes. A study noted that pericardial involvement was associated with lower GLS, in patients with COVID-19 [28]. Reduced GLS might be associated with the severity of myopericardial damages, suggesting that regional longitudinal strain is an important marker, even in asymptomatic COVID-19 patients [28]. Croft et al. study showed that there was no significant difference in terms of LV-GLS in non-survivors compared to survivors, this is likely due to the high prevalence of hypertension in the patients. As previously discussed, hypertension is expected to reduce the LV-GLS [22–24]; thus, difference will be marginal at best. Croft et al. sample size was small, amidst the high prevalence of hypertension; a larger sample size might be needed to demonstrate significant difference. Comorbidities such as obesity, diabetes, hypertension, cardiovascular diseases, and drugs related to them affect COVID-19-related mortality; thus, these variables may confound the analysis, primarily if they are unequally distributed in the two groups [29–33]. GLS has been shown to be an independent predictor of cardiovascular events in patients with diabetes without prior cardiovascular diseases; which thought to be caused by subclinical myocardial systolic dysfunction, reduced GLS was observable even in normotensive and asymptomatic patients [34–36]. Abdominal obesity has also been associated with reduced GLS [37]. Hence, these factors may affect the pooled effect estimate.

The right ventricular strain has previously been shown to be useful for prognostication of patients with ARDS [5, 38]. Inflammation is thought to cause RV overload and damage that resulted in RV failure, which can be assessed by RV-LS [17]. Free wall has a more excellent prognostic value than total RV strain in patients with COVID-19 [16]. One of RV-LS’s advantage is that the performance of RV-LS as a prognostication tool is independent of LV systolic functional index [8]. The included studies uniformly reported that RV-LS was associated with poor outcome with low heterogeneity (11.8%).

Baycan et al. reported that LV-GLS > − 15.2% has an OR of 8.34, area under a curve (AUC) 0.83, sensitivity of 77%, and specificity of 75%, while RV-LS > − 18.45% has an OR of 6.23, AUC 0.77, sensitivity of 72%, and specificity of 66% [16]. Li et al. found that RV-LS at − 23% cutoff was associated with AUC 0.87, sensitivity of 94.4%, and specificity of 64.7% [8]. Croft et al. reported that LV-GLS reduction of > 8.5 showed trend towards increased mortality, but was not independently associated; the unadjusted or adjusted ratio and their corresponding *p* values were not thoroughly reported [6].

### Clinical implications

LV-GLS and RV-GLS are potentially useful in assessing patients with COVID-19. Lower longitudinal strain has been shown to be independently associated with poor outcome. Additionally, cardiac injury is not uncommon and markedly increased the mortality. Hence, routine echocardiography is reasonable and might be useful in patients hospitalized with COVID-19. Considering the time and resources taken to perform echocardiography, the decision should account for individual regions’ capability. In a region with limited resources, routine echocardiography can be performed in COVID-19 with moderate severity or patients at high risk; while the region with greater resources, echocardiography can be performed more liberally. A robust risk prediction model can be made by combining cardiac troponin, natriuretic peptides 10.1136/postgradmedj-2020-137884, electrocardiographic, and echocardiographic findings.

### Limitations

Most of the included studies were retrospective in design, which is a potential source of bias. The data presented by the studies were inadequate to facilitate diagnostic meta-analysis which might be useful in determining post-test probability of patients with high or low GLS. If reported, the cutoff points of LV-GLS and RV-LS were highly polarized. The comorbidities such as hypertension and obesity may affect the analysis; since the number of available studies was small, we cannot perform meta-regression analysis.

## Conclusion

This study shows that lower LV-GLS and RV-LS measurements were associated with poor outcome in patients with COVID-19.

## Data Availability

Data are available on reasonable request.

## Abbreviations

ARDS: Acute respiratory distress syndrome
AUC: Area under a curve
COVID-19: Coronavirus disease 2019
GLS: Global longitudinal strain
LV-GLS: Left ventricular global longitudinal strain
NOS: Newcastle Ottawa Scale
RV-LS: Right ventricular longitudinal strain
SMD: Standardized mean difference
